# Supplementary material for: MetaRibo-Seq measures translation in microbiomes
Source: Nat Commun. 2020 Jun 29;11:3268. doi: 10.1038/s41467-020-17081-z (PMC7324362; doi:10.1038/s41467-020-17081-z)
Supplement: Supplementary file 10 — Supplementary Data 7 [file 41467_2020_17081_MOESM10_ESM.zip › File2/Confidence_High_Taxonomy/409451_out.krona.html]

Javascript must be enabled to view this page.

members
magnitude
magnitudeUnassigned
count
unassigned
taxon
rank

409451\_out

163

superkingdom
2
163

163
976
phylum

class
163
200643

163
171549
order

163
815
family


SRS011134\_contig\_number\_655SRS017191\_contig\_number\_16098SRS018936\_contig\_number\_10887SRS019445\_contig\_number\_12056SRS019601\_contig\_number\_265SRS021219\_contig\_number\_3581SRS023583\_contig\_number\_2239SRS049712\_contig\_number\_18293SRS050941\_contig\_number\_22284SRS053398\_contig\_number\_15329SRS056273\_contig\_number\_710SRS062654\_contig\_number\_359SRS077392\_contig\_number\_contig-100\_523.251970SRS100907\_contig\_number\_contig-100\_1512.63954SRS104975\_contig\_number\_24934SRS146813\_contig\_number\_17589
genus
816
163
16


SRS014235\_contig\_number\_18044
species
246787
1

species

SRS149181\_contig\_number\_92
1
338188


SRS019685\_contig\_number\_9167SRS147346\_contig\_number\_32361
species
818
2

371601
2

SRS052697\_contig\_number\_36713SRS056259\_contig\_number\_10879
species

1263037
2
species

SRS021484\_contig\_number\_12366SRS024625\_contig\_number\_959

124
47678

SRS012273\_contig\_number\_31960SRS013098\_contig\_number\_11296SRS013476\_contig\_number\_41326SRS013951\_contig\_number\_5054SRS013965\_contig\_number\_40239SRS014287\_contig\_number\_3167SRS014313\_contig\_number\_4212SRS014412\_contig\_number\_18858SRS014613\_contig\_number\_8800SRS014683\_contig\_number\_14023SRS014923\_contig\_number\_15474SRS014948\_contig\_number\_20136SRS015264\_contig\_number\_10941SRS016203\_contig\_number\_10307SRS016335\_contig\_number\_1626SRS016541\_contig\_number\_11386SRS016989\_contig\_number\_14202SRS018049\_contig\_number\_3SRS018427\_contig\_number\_80SRS018541\_contig\_number\_9584SRS018623\_contig\_number\_19324SRS018836\_contig\_number\_767SRS018888\_contig\_number\_9697SRS019178\_contig\_number\_4683SRS019267\_contig\_number\_5944SRS019397\_contig\_number\_655SRS019910\_contig\_number\_22410SRS020271\_contig\_number\_1908SRS020328\_contig\_number\_21269SRS022071\_contig\_number\_37651SRS022137\_contig\_number\_12140SRS023715\_contig\_number\_contig-100\_402.121206SRS024132\_contig\_number\_29807SRS024492\_contig\_number\_6693SRS042966\_contig\_number\_11877SRS043001\_contig\_number\_848SRS043667\_contig\_number\_5971SRS043701\_contig\_number\_10119SRS043768\_contig\_number\_2470SRS043841\_contig\_number\_11298SRS044535\_contig\_number\_4974SRS045004\_contig\_number\_2311SRS045244\_contig\_number\_1670SRS045826\_contig\_number\_255SRS046068\_contig\_number\_4106SRS047014\_contig\_number\_20603SRS047044\_contig\_number\_18134SRS047433\_contig\_number\_13411SRS048060\_contig\_number\_13593SRS049995\_contig\_number\_5226SRS050299\_contig\_number\_3888SRS050422\_contig\_number\_24030SRS050752\_contig\_number\_13035SRS050925\_contig\_number\_1672SRS050998\_contig\_number\_17489SRS051031\_contig\_number\_23447SRS052078\_contig\_number\_356SRS053573\_contig\_number\_846SRS054905\_contig\_number\_19851SRS058723\_contig\_number\_1939SRS062427\_contig\_number\_1209SRS063370\_contig\_number\_4290SRS064276\_contig\_number\_10627SRS064557\_contig\_number\_2240SRS064757\_contig\_number\_6056SRS065176\_contig\_number\_226SRS074964\_contig\_number\_5469SRS075821\_contig\_number\_13680SRS075878\_contig\_number\_11433SRS077024\_contig\_number\_9401SRS077086\_contig\_number\_1500SRS077294\_contig\_number\_7578SRS077335\_contig\_number\_1304SRS077454\_contig\_number\_60SRS077502\_contig\_number\_4735SRS077589\_contig\_number\_7753SRS078176\_contig\_number\_6186SRS078242\_contig\_number\_11700SRS078419\_contig\_number\_contig-100\_518.220959SRS078665\_contig\_number\_2494SRS098571\_contig\_number\_262SRS1041039\_contig\_number\_11105SRS1041090\_contig\_number\_7622SRS1041092\_contig\_number\_13153SRS1041118\_contig\_number\_4430SRS1041138\_contig\_number\_12371SRS1041139\_contig\_number\_1626SRS1041141\_contig\_number\_13013SRS1041157\_contig\_number\_5952SRS104311\_contig\_number\_10998SRS104327\_contig\_number\_1292SRS104400\_contig\_number\_44749SRS1054716\_contig\_number\_contig-100\_1733.68291SRS1055022\_contig\_number\_2303SRS1055049\_contig\_number\_3352SRS142542\_contig\_number\_22725SRS142781\_contig\_number\_324SRS142890\_contig\_number\_7953SRS142923\_contig\_number\_5049SRS143181\_contig\_number\_7634SRS143342\_contig\_number\_2425SRS143523\_contig\_number\_517SRS143895\_contig\_number\_46276SRS144183\_contig\_number\_26872SRS144362\_contig\_number\_11916SRS147039\_contig\_number\_1287SRS147557\_contig\_number\_118SRS147614\_contig\_number\_10129SRS148091\_contig\_number\_9711SRS148196\_contig\_number\_40018SRS148253\_contig\_number\_contig-100\_786.93640SRS148319\_contig\_number\_5291SRS148424\_contig\_number\_31574SRS148817\_contig\_number\_11347SRS149244\_contig\_number\_6920SRS149784\_contig\_number\_contig-100\_614.167216SRS892882\_contig\_number\_16136SRS893230\_contig\_number\_2998SRS893279\_contig\_number\_12420SRS893292\_contig\_number\_6692SRS893295\_contig\_number\_4300SRS893300\_contig\_number\_10726SRS893327\_contig\_number\_1030SRS893382\_contig\_number\_5606
species

6
46506
species

SRS017103\_contig\_number\_248SRS077849\_contig\_number\_22199SRS105153\_contig\_number\_2864SRS1055076\_contig\_number\_1858SRS143876\_contig\_number\_22982SRS148253\_contig\_number\_499

species

SRS971276\_contig\_number\_2252
1262737
1

species

SRS105082\_contig\_number\_4591
1
671267

species

SRS017433\_contig\_number\_639
1
817


SRS147977\_contig\_number\_5209
species
469590
1

1841855
1
species

SRS144603\_contig\_number\_11409

1262750
1
species

SRS098827\_contig\_number\_1534


SRS150029\_contig\_number\_1509
species
2292944
1

1262744
1

SRS019381\_contig\_number\_14923
species

species

SRS019030\_contig\_number\_12317
28111
1
